# Eczema in Childhood

**Published:** 1918-09

**Authors:** J. Spencer Davis

**Affiliations:** Dallas, Texas


### ORIGINAL ARTICLES.

### Eczema in Childhood.

J. SPENCER DAVIS, M. D., DALLAS, TEXAS.

Under the title of eczema a great variety of skin diseases have been described.

All undifferentiated skin diseases were placed under this classification, until their recognition, by different observers. There still remains a difference of opinion, however, in regard to whether there is a real difference between a dermatitis and eczema.

One writer very tersely says that the difference between a dermatitis and eczema is that in dermatitis you know the cause of eruption, while in eczema the cause is unknown. Much time and energy has been spent in the search for the cause of eczema, and nearly every drug in the Pharmacopeia, at one time or another, has been used for its treatment.

### VARIETIES.

It may be divided according to:

*I.* *Etiology.*
- (a) Local.
- (b) Constitutional.

*II.* *Age.*
- (a) Infantile.
- (b) Adult.

*III.* *Location of Disease.*
- (a) Scalp.
- (b) Face.
- (c) Intertrigo between the thighs and around the joints of the body, etc.

*IV.* *Character of Lesions.*
- (a) Paptular.
- (b) Vesicular, etc.

Eczema is a very frequent disease of childhood and is more prevalent than all the others combined. In the infantile type the child is very frequently fat and well nourished.

### I. ETIOLOGY.

Eczema is always secondary to some local or constitutional condition. The peculiar constitutional condition which predisposes to eczema, has been described under the title of Exudative Diathesis, by Czerny.

Children affected with this condition are very much predisposed to inflammations of the respitory and gastro intestinal tracts, and exudation of the skin. These children will have frequent colds or diarrhea accompanied by rash on the skin. They have a general glandular enlargement and, notwithstanding they are well nourished, are predisposed to tuberculosis. They have a very low tolerance for fat, are frequently constipated, and pass dry putty colored stools. The urine in such cases has an ammonacal odor.

X-ray and percussion frequently show a much enlarged thymus gland. Some cases will have attacks of cyanosis preceded by violent spells of crying.

### FOOD.

Certain varities of food produce eczema, and whatever eaten produces the characteristic eruption in a short time. The fats and carbohydrates are the ones most often responsible for producing the rash. Protein of food is not a direct cause of eczema, but sometimes starts an eruption on the skin which becomes eczematous.

We have now a means of testing for these Protein food idiosyncrasies. A number of supply houses prepare these foods in a pure form, and all that is necessary is to make a slight abrasion on the skin, apply a small quantity of food to be tested, and a drop of tenth normal Sodium Hydroxide.

Where the child has idiosyncracy to the food in a few minutes we get an area of redness and skin looks elevated, very much like a wheal. This usually persists for a few hours.

Some writers think that fat produces a moist eczema, while starch produces the dry scaly type. Kerley groups these together under the heading of Carbon Incapacity, and points out that the eczema may be the only manifestation of food disturbance. It appears to make very little difference whether the fat and sugar comes from breast milk or cows milk.

In eczema of children we see clinically two types (a) Dry; (b) Moist, and while both types may be present at the same time in one individual, yet usually the eruption, although in different locations, is of one type.

The dry type of eczema is supposed to be connected with disturbances of utilization of Carbohydrates especially starch.

The Moist type is usually due to faulty metabolism of fats and sometimes of sugar. After the eruption has been started a super imposed infectococcus and the picture is entirely changed.

The eruption on extremities occurs for the most part on limbs.

### INFECTION.

In older children eczema is occasionally produced by localized infectious processes such as occurs around decayed teeth, throat, and sinuses of the head. These cases are very resistant to treatment, until the infectious process is eradicated.

I have frequently noted a type of eczema in congenital syphilitics which could not be relieved until the anti-syphilitic treatment had been carried out vigorously.

Local infections of the skin may produce an eczema also but it is usually corrected by the means used in treating the infections and requires no special discussion.

### TREATMENT.

In treating this condition in children the importance of regulation of diet can not be too strongly emphasized. It is of far more importance to many cases than local treatment, and frequently no headway in checking the eruption, can be made until the diet has been changed.

### INTERTRIGO.

It is well to keep in mind, however, that this eruption occuring between the thighs and region covered by the napkin, may have a local as well as a dietary cause. Frequently the napkin will not be sufficiently rinsed after being washed and a part of the soap will dry in the napkin, as soon as the child urinates, there is a soap poultice in contact with the skin which produces the rash.

Other cases arise as a result of putting on napkins, which have simply been dried without washing after being soiled with urine. This pernicious habit is now fast disappearing. The correction of the trouble in these cases requires no comment, but there is a type of intertrigo due to ammonical urine passed directly from the bladder. All of these cases are due to the excessive ingestion of fat in the food. This excess of fat is first digested and neutralized by fixed alkalines Sodium and Potassium but as soon as these reach a low level the ammonia is called upon and the ammonia salts thus formed escapes in the urine and give rise to the skin inflammation. In these cases, the napkin should be removed as soon as soiled with urine, the parts should be bathed with water and after drying a bland protective ointment such as zinc oxide should be applied thoroughly. The quantity of fat in the food should be reduced.

In breast fed children this can be accomplished by lengthening the nursing interval, decreasing the meat and rich foods in mothers diet, and increasing her exercises.

In most instances the child should not nurse oftener than every four hours. In bottle fed children the milk could be skimmed or buttermilk feeding substituted.

Where urine is very acid, alkalies should be administered. Sodium Bicarb or citrate may be used to alkalinize the patient.

In moist types there is usually a great deal of itching and the child tries continuously to scratch or rub the parts. Local applications can be used to relieve this itching. One of two types as usually employed: (a) Lotions such as calamine lotion. (b) Ointments such as zinc oxide.

Where the eruption occurs on the face it is usually necessary to have a patient wear a mask, such as the Herter Mask, and a sort of straight jacket supervised to prevent the scratching of the skin. The writer has frequently used a mailing tube, such as are used in sending calendars, to put over the arms to prevent the arm from bending and a safety pin can be used to fix the arm to the bed clothing.

In the dry scaly type of eczema, in addition to regulating the diet thyroid extract should be administered.

